# Composition and Seasonality of Membrane Transporters in Marine Picoplankton

**DOI:** 10.3389/fmicb.2021.714732

**Published:** 2021-09-28

**Authors:** Åke Hagström, Ulla Li Zweifel, John Sundh, Christofer M. G. Osbeck, Carina Bunse, Johanna Sjöstedt, Bärbel Müller-Karulis, Jarone Pinhassi

**Affiliations:** ^1^Centre for Ecology and Evolution in Microbial Model Systems – EEMiS, Linnaeus University, Kalmar, Sweden; ^2^Swedish Institute for the Marine Environment, Gothenburg University, Gothenburg, Sweden; ^3^Department of Biochemistry and Biophysics, National Bioinformatics Infrastructure Sweden, Science for Life Laboratory, Stockholm University, Solna, Sweden; ^4^Helmholtz Institute for Functional Marine Biodiversity at the University of Oldenburg (HIFMB), Oldenburg, Germany; ^5^Department of Biology, Aquatic Ecology, Lund University, Lund, Sweden; ^6^Baltic Sea Centre, Baltic Nest Institute, Stockholm University, Stockholm, Sweden

**Keywords:** bacterial succession, membrane transporter traits, substrate uptake, toxin secretion, biogeochemical indicator

## Abstract

In this study, we examined transporter genes in metagenomic and metatranscriptomic data from a time-series survey in the temperate marine environment of the Baltic Sea. We analyzed the abundance and taxonomic distribution of transporters in the 3μm–0.2μm size fraction comprising prokaryotes and some picoeukaryotes. The presence of specific transporter traits was shown to be guiding the succession of these microorganisms. A limited number of taxa were associated with the dominant transporter proteins that were identified for the nine key substrate categories for microbial growth. Throughout the year, the microbial taxa at the level of order showed highly similar patterns in terms of transporter traits. The distribution of transporters stayed the same, irrespective of the abundance of each taxon. This would suggest that the distribution pattern of transporters depends on the bacterial groups being dominant at a given time of the year. Also, we find notable numbers of secretion proteins that may allow marine bacteria to infect and kill prey organisms thus releasing nutrients. Finally, we demonstrate that transporter proteins may provide clues to the relative importance of biogeochemical processes, and we suggest that virtual transporter functionalities may become important components in future population dynamics models.

## Introduction

Direct interactions between marine microorganisms and the pool of dissolved organic matter and nutrients *via* the cell membrane was early on the subject of studies in microbial ecology (Azam and Hodson, 1977; Hagström et al., 1984). Until the era of high throughput sequencing, the uptake of resources was primarily studied by proxy measurements, providing uptake rates through for example radioactively labeled substrates [(Hobbie and Wright, 1965; Azam and Hodson, 1981) and references therein]. Today it is broadly recognized that the most oceanic biogeochemical processes, including key reactions such as fixation of carbon, nitrogen, and the respiratory dissimilation of organic matter, are dominated by microbial communities (Azam and Worden, 2004). Uptake of dissolved organic and mineral nutrients is achieved through transporter proteins operating across the cell membrane, a feature conserved in all domains of life (Ren and Paulsen, 2005). These transporter proteins have been categorized based on their function and molecular structure, with the following four mechanisms representing the majority of known transporters: (1) porins and channels, (2) electrochemical potential-driven transporters, (3) primary active (energy dependent) transporters, and (4) group translocators (Saier et al., 2016). Transporters in these broad categories often contain a substrate binding domain or protein that provides the substrate specificity of the transporter (Berntsson et al., 2010), thus allowing an identification of the substrate specificity. The molecular structure and mechanism of representatives of each of the transporter classes have been extensively studied and can be reviewed in any current biochemistry textbook. Yet, while the biochemical knowledge regarding these membrane proteins is solid, the ecological implications of the transporter proteins for microorganisms in their natural environment are much less studied.

Through meta-omics, including metagenomic and metatranscriptomic surveys in time and space, this situation has changed and data sets that reveal both potential substrate transformations and links within and between various metabolic pathways in different organisms are now emerging. Importantly, these studies reveal a differentiated expression of transporters between geographic locations, between environmental conditions, such as phytoplankton bloom versus non-bloom conditions, and in particle-associated compared to free-living microorganisms (Klindworth et al., 2014; Satinsky et al., 2014). In the North Sea, temporal succession of bacteria and the associated expression of transporters have been tentatively linked to diatom blooms, implying a substrate driven succession of the bacterioplankton community (Teeling et al., 2016). This indicates the potential for obtaining ecological insight from analyzing transporter dynamics across seasonal cycles in the marine environment. However, the technical bottleneck of transporter detection in omics dataset remains a main issue since the accuracy and completeness of databases used for gene annotation still can be improved. Consequently, results on microbial functions derived from metagenomes in natural environments must be interpreted with caution.

In the Baltic Sea, sampling at the Linnaeus Microbial Observatory (LMO) during the last decade has generated a long-term time series of omic’s data (Alneberg et al., 2018a; Bunse et al., 2019). Using the omic’s data set, we analyzed the taxonomic distribution of membrane transporters, their abundance, and putative substrate recognition, in order to answer the following three interrelated questions that could provide knowledge on transporter proteins in the context of aquatic microbial ecology: (i) does microbial succession correlate with the presence of different importer traits, i.e., transporter proteins directing the uptake of organic and mineral nutrients, (ii) does the distribution of transporter proteins involved in virulence (e.g., toxin secretion) imply that marine bacteria engage in predatory life strategies to obtain nutrients (Persson et al., 2009; Hagström et al., 2017), and (iii) do transporter proteins provide clues to prevalent biogeochemical processes in the marine environment? We hereby reasoned that sampling across seasons at the LMO station in the Baltic Sea would allow insights into the potential partitioning of resources among components of the microbial community viewed from the point of transporter functionality.

## Materials and Methods

### Sampling

**Figure 1:**
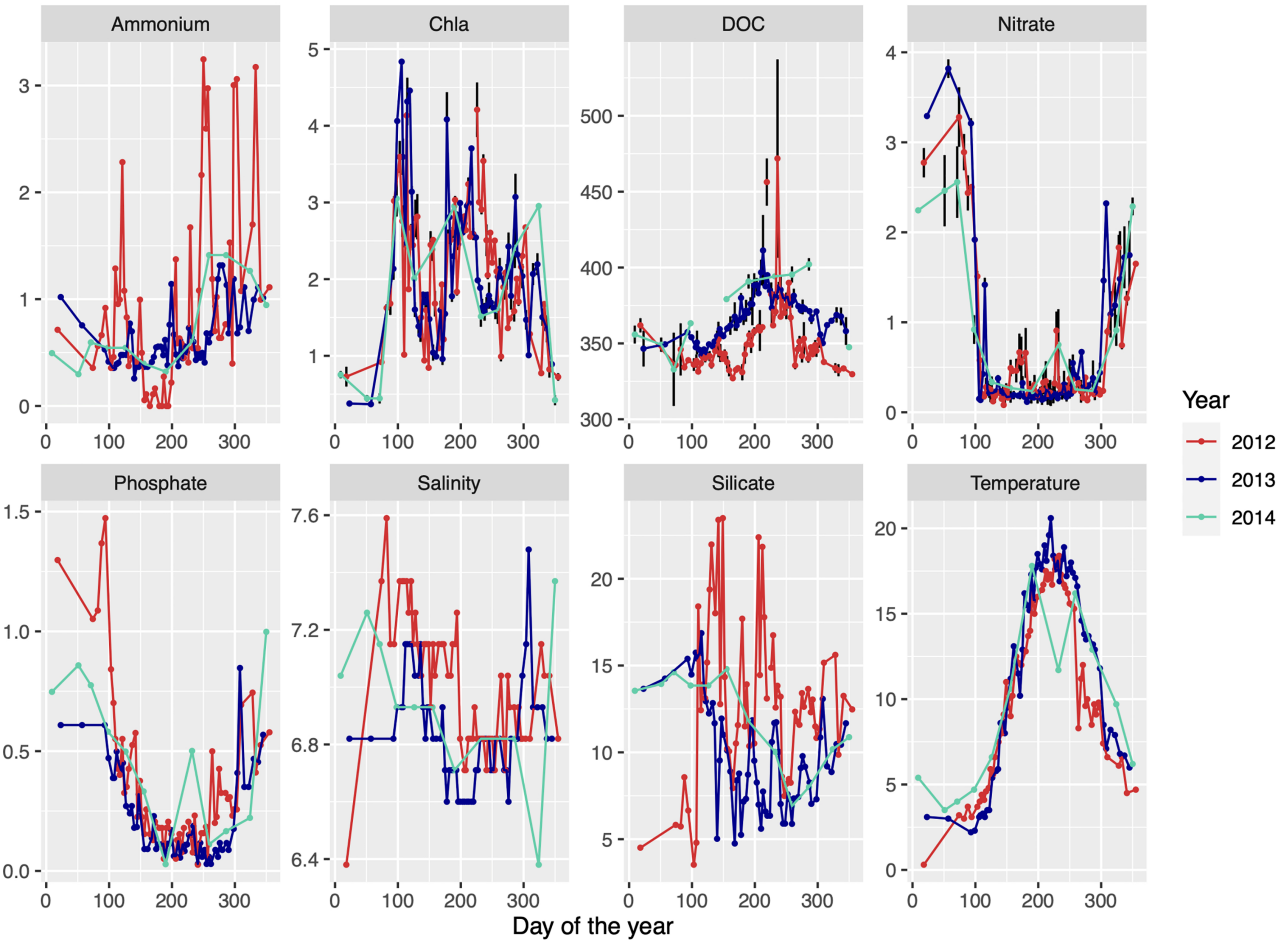
Environmental data of ammonium (μmolL^−1^), chlorophyll a (μgL^−1^), dissolved organic carbon (DOC; μmol C L^−1^), nitrate (μmolL^−1^), phosphate (μmolL^−1^), salinity (PSU), silicate (μmolL^−1^), and temperature (°C) over three consecutive seasons throughout 2012 until 2014 at the Linnaeus Microbial Observatory (LMO) sampling site, off the island of Öland, Sweden, in the southwest Baltic Proper. Data are redrawn from Lindh et al. (2015) and Bunse et al. (2019) and denote averages of technical replicates.

Surface water samples were collected from 2m depth at the Linnaeus Microbial Observatory (LMO), 10km east off the island Öland, Sweden, in the central Baltic Sea (56°55′0.51.24′N 17°3′38.52′E) using a Ruttner sampler. Water was collected in 10L polycarbonate containers and transported within 1h to the laboratory at Linnaeus University in Kalmar. Samples for abiotic and biotic variables for 2012–2014, other than DNA and RNA (described below), were taken as published previously in Lindh et al., 2015; and Bunse et al., 2019. Briefly, temperature and salinity were measured directly on site. Samples for chlorophyll *a* (Chl *a*) and nutrients were processed in the laboratory and data depicted in Figure 1 display averages of technical replicates.

A total of 37 DNA samples for metagenome sequencing were collected between March and December 2012, by filtering seawater (around 3L) first through a 3.0μm polycarbonate filter and then collecting microbial biomass onto a 0.22μm filter (GP, Sterivex, EMD Millipore) Legrand et al., 2015. Filters were flash frozen in liquid nitrogen following addition of Tris-EDTA buffer (pH 8.0), and stored at -80°C until DNA extraction (Hugerth et al., 2015). For technical reasons, four of the samples were not included in our analysis, resulting in a total of 33 samples for the current study.

Samples for metatranscriptomics sequencing were collected on 25 occasions during the period 2012 to 2014, although only four samples from 2012 coincided in time with the metagenomic samples. For RNA, approximately 8L of seawater were prefiltered through a 3.0μm, 142mm diameter polycarbonate filter (Maine Manufacturing, LLC, United States), followed by a 0.2μm, 142mm diameter polycarbonate membrane disk (GVS life science, United States) in less than 20min. The 0.2μm filters were then added to 15ml RNase/DNase-free tubes with 2ml RLT buffer (QIAGEN) containing 0.01% Beta-mercaptoethanol, and flash frozen in liquid nitrogen and stored at -80°C until further RNA extraction processing.

### Nucleic Acids Extraction and Sequencing

DNA extraction from the sterivex filters was done by a phenol-chloroform protocol, including a 30min lysozyme digestion step at 37°C and a proteinase K digestion overnight at 55°C, as detailed in Boström et al. (2004). Details on sequencing of the metagenomic samples are as in Buchan et al. (2014). Briefly, for each sample, 2–10ng of DNA was prepared with the Rubicon ThruPlex kit (Rubicon Genomics, Ann Arbor, Michigan, United States) according to the manufacturer’s instructions. Cleaning was done with MyOneTM superparamagnetic beads (Invitrogen, Carlsbad, CA, United States). DNA libraries were sequenced at SciLifeLab Stockholm, Sweden, on a HiSeq 2500 (Illumina Inc., San Diego, CA, United States) using a 2×100bp paired-end reads setup.

Extraction of RNA was performed according to a protocol adopted from Poretsky et al., 2010. Briefly, samples were thawed and mechanical lysis of cells was done twice using powersoil beads (MOBIO RNA powersoil beads tubes) for 10min. After centrifugation (5,000*g*), the supernatant was collected without carryover of beads and transferred to a new tube. One volume of 70% ethanol was added and tubes were gently mixed five times. The extraction was then continued using RNeasy mini columns (RNeasy mini kit, QIAGEN) according to manufacturer’s instructions. The samples were then cleaned from DNA using Turbo DNA-free kit (AMBION) and mRNA was linearly amplified using Message Amplification II bacteria kit (AMBION) according to the manufacturer’s protocol. Sequencing was completed at SciLifeLab Stockholm, Sweden, on a HiSeq2500 (Illumina Inc., San Diego, CA, United States) with a 2×126bp paired-end reads setup in RapidHighOutput mode.

### Reference Metagenome Assembly and Annotation, Sequencing Read Preprocessing, and Mapping

The current analysis of metagenomes and metatranscriptomes was made extensive use of the Baltic Sea Reference Metagenome (BARM; Alneberg et al., 2018b). BARM is a metagenomic reference assembly generated from 81 water samples collected in the Baltic Sea, and the LMO metagenomic samples studied here (collected in 2012) were among the samples used to construct the BARM assembly. Details on identification of open reading frames (ORFs), and the functional and taxonomic annotation of ORFs are given in Alneberg et al., 2018b. ORFs in BARM have been functionally annotated with COGs, PFAMs, and TIGRFAMs. Moreover, ORFs have been given a taxonomic assignment by querying protein sequences against the GenBank nr database using diamond (v0.8.26), followed by assignment of a lowest common ancestor from the list of top-scoring hits.

As a first step in the current analysis of transporter genes, the entire set of metagenome sequencing reads was preprocessed as in Alneberg et al., 2018b, including adapter removal (universal Illumina adapters with default settings) and quality trimming (Phred score>15) with cutadapt, keeping read pairs of at least 31bp in length. For the metatranscriptomic samples, in turn, adapters were removed with cutadapt (v1.8.0; Martin, 2011) using the universal Illumina adapters and default settings. This was followed by quality trimming using sickle (v1.210) with default settings. Finally, rRNA reads were removed using SortMeRNA (v2.0; Kopylova et al., 2012) with default settings and all rRNA databases.

Next, to quantify the distribution of sequencing reads among the total set of ORFs in BARM, the preprocessed metagenomic and metatranscriptomic reads were mapped to the BARM reference assembly using bowtie2 (v2.2.6; Langmead and Salzberg, 2012) with default settings except for the use of the “–local” parameter. This was followed by removal of duplicate reads using picard (v1.118), counting of mapped reads using htseq (v0.6.1; Anders et al., 2015) and normalization using the “TPM” method as described in Wagner et al., 2012.

Although taxonomic assignments were made for individual ORFs, these assignments were then used in a voting strategy to assign a lowest common ancestor to the corresponding contigs. This is similar to the method used by, e.g., CAT, but is based on alignment fraction and percent identity of hits and not directly on bit scores, as described in Alneberg et al., 2018b. The assigned contig taxonomy was then propagated to all ORFs on that contig. The abundances of all ORFs obtained by mapping and counting the reads from the metagenomic and metatranscriptomic samples onto the BARM reference assembly constituted the basic dataset, consisting of quantitatively determined taxonomically and functionally annotated ORFs, from which we proceeded to further identify and annotate membrane transporters (to ultimately obtain a curated set of quantified and identified transporters, see next section).

### Compilation and Curation of Membrane Transporter Data Set

To obtain a curated identification of membrane transporters among the ORFs in BARM, beyond the direct annotations in BARM obtained by individual PFAMs, TIGRFAMs, or COGs, we screened the PFAM, TIGRFAM, and COG databases (as of March 2018) for protein families related to transporter proteins using text searches (e.g., “transport,” “efflux,” “uptake,” “symport,” “antiport.” See full pattern at https://github.com/johnne/transporters/wiki/1.-Identifying-protein-families). This search identified 528, 464, and 443 transporter protein families in the PFAM, TIGRFAM and COG database, respectively. These protein families were merged into transport clusters using cross-reference information for 25,552 reviewed entries in the Uniprot database^1^ (as of December 2017), and all operons defined in the OperonDB^2^ database. That is, if several protein families were found on the same Uniprot entry or in a defined operon, they were clustered to represent the same transporter. In the present study, we hereafter refer to membrane transporters as the full set of subunits that are jointly necessary to assemble a functional transporter (and not individual transporter genes). In our method, each protein family (be it a TIGRFAM, COG, or PFAM) can be viewed as a “node” and every time we found a reviewed Uniprot entry annotated with more than one protein family each unique pair-wise combination of those protein families defined an “edge”. This, together with information in OperonDB, proved very efficient in linking together information in the different protein family databases. However, generic protein families, such as the ATP binding domain PF00005, which are annotated on diverse sets of proteins have a lot of edges and can lead to inflated transporter clusters. We therefore ignored protein families with more than six edges in order to prevent inflation. To prevent inflation of transporter clusters, broadly defined protein families, such as the PF00005 ABC transporter domain, were ignored by setting a maximum number of allowed outgoing “edges” to six. Scripts and methods for identifying and clustering transporters are found on GitHub at https://github.com/ChristoferLNU/transporters/.

The full list of transporter proteins and their COG, TIGRFAM, and PFAM identifiers is available at https://github.com/ChristoferLNU/transporters/blob/master/results/transport-clusters.2017_12.tab. Having compiled the list of transporter proteins and the corresponding protein family identifiers of the constituent proteins, we used these identifiers to locate ORFs in the BARM assembly that represented the membrane transporters. Together with the output from the mapping of the omics reads toward the ORFs in BARM, we thus quantified the relative abundance of each transport cluster in the studied samples.

### Substrate Categorization of Transporter Proteins

Identified transporter clusters were further annotated with putative substrates using information in the Gene Ontology and TIGRFAM roles databases. Where information in these databases could be linked to protein families contained in each transporter cluster that information was used to assign a putative substrate to the corresponding transporter cluster, using manual curation where the TIGRFAM role was given precedence. Transporter clusters that could not be linked to these databases were not considered for further analysis.

For each of the transporter proteins listed in the Supplementary Table S1, the information in the TIGRFAM summary page for a given protein family was manually inspected and the proposed gene name and published references were collected. The original literature references were identified in the NCBI library together with recent literature (year 2015 and later). These studies were checked to ensure that the gene in question was associated with the cited function proposed in the database summary page. Any further information on the specific transporter function, such as metal transport or virulence involvement, was noted. In case no literature reference was given in the summary page, the gene name was searched in the NCBI “Protein Family Models” resource, to make sure that the most recent update of the function associated with the gene name still corresponded to the function cited in the TIGRFAM summary page.

Based on the protein family description, the transporter clusters were divided into two main groups importers and exporters to facilitate the analysis, although a clear distinction regarding the direction of transport was sometimes difficult to establish, e.g., for metal ion transporters. The importer proteins could be further subdivided, based on the putative substrate recognition of the suggested transporter clusters, into one of nine substrate categories: (1) amino acids (AA) and peptides + NH_3_, (2) carbohydrates, (3) nucleosides, (4) anions, (5) cations, (6) nitrate, (7) urea, (8) phosphate, and (9) phosphonate. With respect to transporter function, the anion group represents mechanisms for the uptake of negatively charged ions, such as sulfate and molybdate, but also importantly small charged organic molecules. The cation group is directed toward metal-ions, such as iron, cobalt, magnesium, nickel, and zinc, but also includes transporters for the uptake of organic molecules, in particular large and complex molecules. Analyses conducted for this study are available online at https://github.com/ChristoferLNU/transporters/tree/master/article as a set of python notebooks.

### Data Availability

Data generated in this study can be found through the following DOI link https://doi.org/10.6084/m9.figshare.c.4508144.v2.

## Results

### Overall Characteristics of the LMO Omics Datasets

A compilation of 3years (2012, 2013, and 2014) of biological and environmental data from the LMO station is presented as background information in Figure 1. The Baltic proper is a temperate sea area where the surrounding land areas are frozen and the available sun light is quite low during the winter period, and thus the growth period is only about 10months and not a full year. To allow comparisons with other sea areas, the growth periods are named spring, summer, and autumn throughout. The winter period, which is largely missing from the omics results, is characterized as a non-growth period (Figure 1) with high levels of nitrate, low levels of Chla, and low temperature (Hagström et al., 2001). The metagenomic dataset (hereafter referred to as MG) comprised 33 samples obtained on a biweekly basis, from spring to early winter in 2012 at the LMO station. The metatranscriptomic dataset (hereafter referred to as MT) was sampled at the same site as the MG dataset, but on a monthly basis from early summer of 2012 to the fall of 2014. Four out of 58 samples have MG and MT data collected on the same dates (May 16, 2012; June 13, 2012; August 13, 2012; and December 20, 2012). Thus, unless dates are specified, averages from the growth periods were considered when comparing the MG and MT datasets.

Throughout 2012, the MG dataset was comprised of roughly 75% bacterial, 18% pico-eukaryotic, and 6% viral protein coding sequences (Table 1). Archaeal sequences accounted for on average 0.4%. The high number of viral sequences is noteworthy since it may be argued whether or not transporter proteins are common or rare in viruses or even if they have a function (Greiner et al., 2018). At the same time, phage dynamics in the marine environment is seen to be increasingly important (Breitbart et al., 2018). We decided to report the ORFs associated with virus transporter proteins in Table 1 as an information to others but have not addressed these issues further.

**Table 1:** Proportion of transporter proteins in selected taxon of the metagenomic and metatranscriptomic datasets.

Taxon	Metagenomics (MG), 33 samples collected during the 2012 growth period				Metatranscriptomics (MT), 25 samples collected during the 2012, 2013, and 2014 growth periods			
	mean (%)	max (%)	min (%)	Std^*^	mean (%)	max (%)	min (%)	Std^*^
Archaea	0.44	2.95	0.09	0.53	0.19	1.73	0.01	0.34
Bacteria	75.43	85.96	56.86	7.65	54.83	92.3	4.58	18.73
Picoeukaryota	17.71	39.5	7.11	7.99	43.61	95.36	6.63	19.33
Unclassified	0.07	0.13	0.03	0.03	0.02	0.06	0.0	0.02
Viruses	6.34	16.51	1.15	3.16	1.35	12.48	0.04	2.5
Cyanobacteria	2.57	6.45	0.93	1.35	4.97	27.72	0.34	5.65

* *Std represents standard deviation*.

*Sample dates same as for Figure 7*.

**Figure 2:**
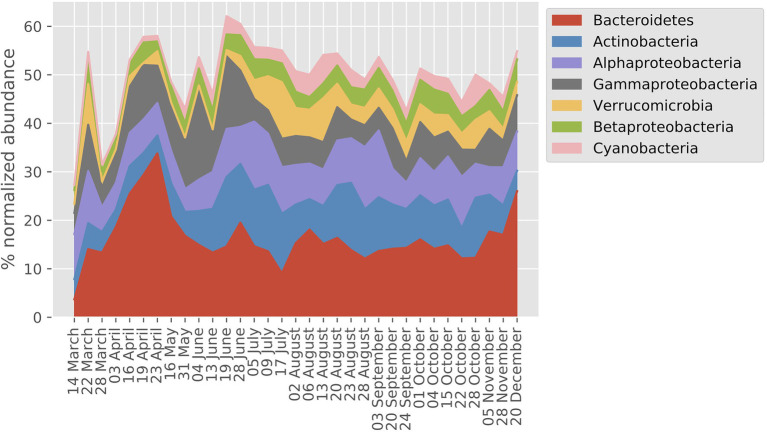
Prokaryote taxonomic profile derived from normalized abundance in the LMO metagenome (MG) dataset (year 2012). The percentage of the seven phyla/classes with highest mean across sampling dates are shown.

The seven most abundant major bacterial taxa (Phylum/Classes) together accounted for between 40 and 60% of the MG dataset on an annual basis (Figure 2). Overall, *Bacteroidetes*, *Actinobacteria*, and *Alphaproteobacteria* showed the highest relative abundances, and most taxa showed a two- to three-fold range of variation in abundance during the year. On average, 54% of the transcripts were bacterial, 44% picoeukaryotic, and 1% viral whereas archaeal transcripts made up 0.2%.

### Number of Identified Membrane Transporters

In the BARM co-assembly, a total of 66,029 ORFs (corresponding to ~1.0% of total 6,757,106 ORFs) were identified as membrane transporters or subunits thereof. The number of transporter ORFs detected in the MG and MT datasets by read mapping was 39,634 (0.85% of total ORFs) and 20,166 (0.43% of total ORFs), respectively.

The assembled transporter ORFs were classified into 1,149 membrane transporter clusters (Table 2), based on at least one TIGRFAM protein family being identified as having transporter functionality. These figures refer to the number of ORFs identified in the assemblies. The abundance of transporters is reported as normalized count data and was calculated by mapping reads to the assemblies. The full list of transporters can be found in Supplementary Table S1. The number of annotated transporter proteins totaled 338 (Table 2). Of these, a total of 124 transporter proteins reached a relative abundance of >0.5% of the total abundance of the identified transporter proteins during at least one sampling occasion seen in the MG and MT datasets and are hereafter referred to as “abundant” transporters. A total of 59 abundant transporters were identified as nutrient importers and almost as many abundant transporters (46 proteins) were identified as responsible for export functions. Among the exporters, 21 were related to toxin secretion. Metal transporters and a group of “Other” amounted to 19 proteins that were addressed in order to provide a comprehensive description of the transporter proteins (Table 2).

**Table 2:** Number of unique transporter proteins in each different transporter category, with reference to the total number of transporter protein clusters with association to identified protein families.

Number of identified transporter protein clusters	1,149	Breakdown of transporter protein clusters				
Number of transporter protein cluster linked to TIGR protein families	338	Nutrient importer proteins	Exporter proteins, utility secretion	Exporter proteins, toxin secretion	Other transport proteins	Metal transport proteins
Number of transporter proteins that amounted to >0.5% in either the MG or MT datasets	124/equals	59	25	21	10	9

### Importers

As defined in the Transporter Classification Database (TCDB; Saier et al., 2016), the majority of the importers belonged to two groups: the electrochemical-potential driven transporters type 2A (43%) and the primary active transporters type 3A (42%) that require P–P bond hydrolysis and represent the most energy demanding transport system. Carbohydrate and nucleoside transporters were primarily found among the former type while urea and phosphorus related to transporters were primarily found among the latter type. The remaining 15% of importers were distributed among the following seven TCDB groups: 1A, a-type channel 5%; 1B, b-strand porins 2%; 2C, Ion-gradient driven energizer 1%; 3D, Oxidoreduction-driven transporters 4%; 4A, Phospodriven group translocator 2%; 4B, Nicotinamide ribonucleoside uptake transporters 2%; 9A, Recognized transporters of unknown biochemical mechanism 1%.

### Importers and Substrate Recognition

**Figure 3:**
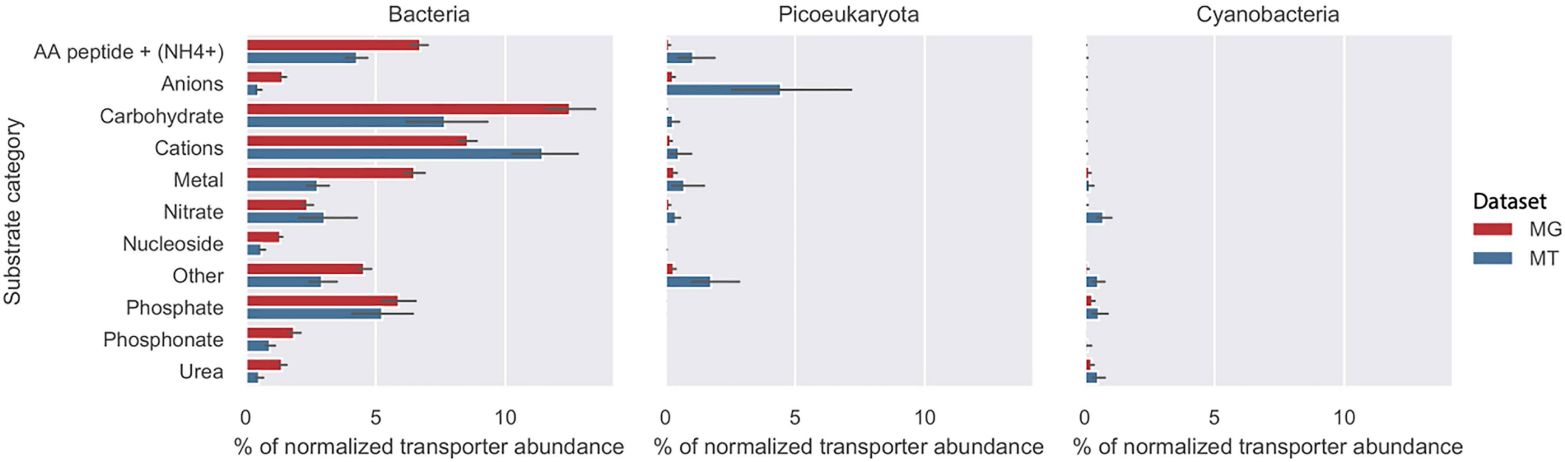
Substrate recognition by transporter proteins in the LMO metagenome (MG) red bars and metatranscriptome (MT) blue bars. Mean total abundance of each substrate category during the 2012 growth period (10months) for bacteria, picoeucaryotes, and cyanobacteria as a proportion of total transporter abundance. The abundance of transporter proteins genes associated with cyanobacteria was subtracted from the Bacteria fraction.

The abundance of importer proteins of bacteria, picoeukaryotes, and cyanobacteria in relation to nine substrate categories are shown in Figure 3. In both the MG dataset (2012 biweekly) and the MT dataset (2012–2014 monthly), the primary contributors to transporter abundance and transcription within all substrate categories were heterotrophic bacteria. The most abundant transporters were associated with four substrate categories: cations, carbohydrates, amino acids/peptides and ${\mathrm{NH}}_{4}^{+}$, and phosphate. In addition to the nine substrate categories, we defined a group as “others” that contained less common transporters, such as iron complexes and vitamin B12 (Schauer et al., 2008; Figure 3). More information on these genes can be found in Supplementary Table S1. Cyanobacteria, such as *Synechococcus* and picoeukaryotes, contributed only minimally to the abundance of transporter proteins in the MG and MT dataset.

**Figure 4:**
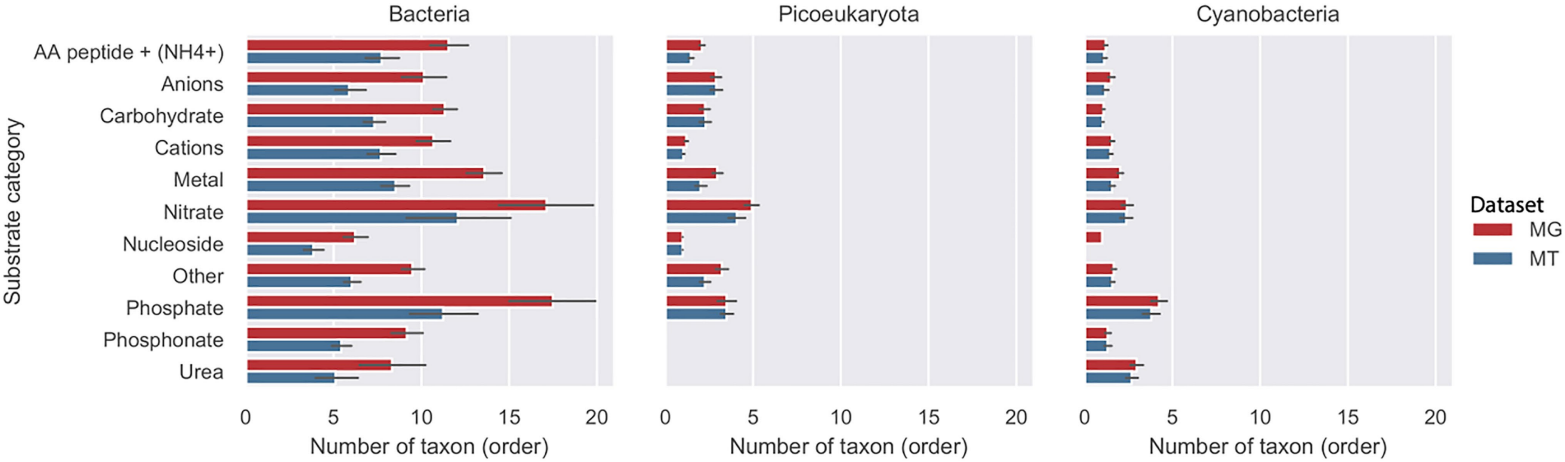
Diversity of transporters in three taxonomic groups during the 2012 growth period (10months). Number of taxa (order) at rank order shown per putative substrate category for bacteria, picoeucaryotes, and cyanobacteria in the LMO metagenome (MG) shown with red bars and metatranscriptome (MT) shown with blue bars.

The average number of taxa (orders) for the three groups bacteria, picoeucaryota, and cyanobacteria associated with each substrate category is shown in Figure 4. In the metagenome, the results for bacteria demonstrated that in most substrate categories the importers were accounted for by around 10 orders during the 2012 growth period (10months). Exceptions were the phosphate and nitrate categories that were found in approximately twice as many orders, and the nucleoside category that was accounted for by nearly half as many orders. In the MT data, the transcripts showed a similar relative distribution of active importers compared to the genome data, but the number of orders in each category was about 25% lower compared with the MG data set. For cyanobacteria and picoeukaryotes on average, only two orders were associated with each of the substrate categories.

There was no reason to expect that gene abundance would be similar to the number of transcripts but it was seen that the relative abundances in the MG, and the transcripts in the MT datasets of transporter proteins within the different substrate categories were similar, with the exception being cations that showed higher relative transcription (Figure 3).

A prerequisite to use specific genes, for instance microbial transporter genes, as indicators of environmental status, will be that the abundance of the genes is accompanied by a corresponding activity (transcription). Potential correlations between transporter abundance and transcription were further examined in the MG and MT datasets for the four sampling dates when both MG and MT data were available, focusing on the four orders *Flavobacteriales*, *Cellvibrionales*, *Sphingobacteriales*, and *Mamiellales* that had more than six substrate categories that could be tested (Table 3). This comparison indicated that 17 of the 29 individual correlations had correlation coefficients >0.6. For example, in *Cellvibrionales*, both carbohydrate importers and phosphate importers were seen to correlate in the metagenome and transcriptome datasets. In *Sphingobacteriales*, the strongest correlations were found for anions, nucleosides, cations, and amino acids/peptide importers (Table 3).

**Table 3:** Correlation between metagenome (MG) abundance of transporter clusters and metatranscriptomic (MT) expression of transporter clusters at four sampling dates, see Section “Materials and Methods.”

Substrate category	*Cell-vibrionales*	*Flavo-bacteriales*	*Mamiellales*	*Sphingo-bacteriales*
AA peptide + ${\mathrm{NH}}_{4}^{+}$	0.70^*^	0.55	0.93^*^	0.94^*^
Anions	0.41	–	0.96^*^	1.00^*^
Carbohydrate	0.90^*^	0.57	0.68^*^	0.81^*^
Cations	0.60^*^	0.22	0.55	0.97^*^
Metal	0.47	0.68^*^	0.95^*^	0.98^*^
Nitrate	0.54	−0.28	0.54	−0.17
Nucleoside	−0.05	0.95^*^	–	0.99^*^
Phosphate	0.89^*^	−0.44	–	–

* *Coefficient of correlation>0.6*.

*The correlation coefficient is presented for each of the clusters affiliated to Flavobacteriales, Cellvibrionales, Mamiellales, Sphingobacteriales, and 8 different substrate categories*.

### Number of Importer Types in Different Microorganism Taxa

**Figure 5:**
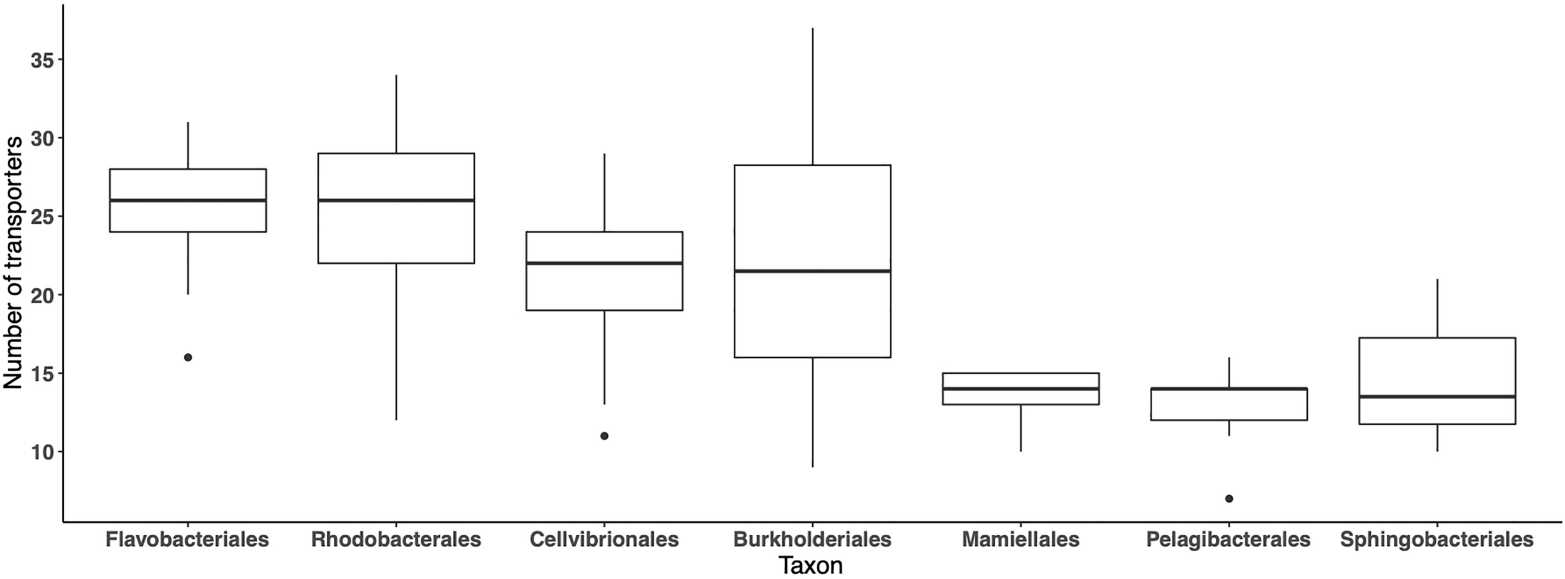
Number of different transport protein clusters for each of the seven most abundant orders, across the LMO 2012 metagenome dataset. For each order, the value at a specific sample point is only shown if the sum of normalized transporter abundance for that order was greater or equal to the 90% quantile calculated for all orders in the sample.

In the MG dataset, 59 abundant importers have been identified in 185 bacterial orders, while in the MT dataset, 131 orders were represented in the same 59 abundant importers, indicating that the importer genes were expressed by ~70% of the taxa comprising the 59 abundant importers. We next analyzed the average number of different importer types associated with different taxa (orders) during the 2012 growth period, in the MG and MT datasets, where >500 reads mapped to the set of 59 abundant transporters. On average, around 20–25 importers were detected across samples in the orders *Cellvibrionales*, *Flavobacteriales*, *Rhodobacterales*, and *Burkholderiales*, although with high variability around the averages (Figure 5). In contrast, <15 transporters were associated with the *Pelagibacterales*, which are oligotrophic bacteria characterized by small cell sizes and with streamlined genomes (Giovannoni et al., 2005). Similarly, relatively few different transporters were found in the order *Sphingobacteriales* (Bacteroidetes) and the green algae order *Mamiellales*. In the MT dataset, the highest number of transporter proteins was found in the bacterial orders *Flavobacteriales* and *Cellvibrionales* with between 15 and 20 importers per sample (data not shown). Collectively, these findings show that there are pronounced differences in the number of importers between orders.

### Importer Abundance Versus Substrate Category

**Figure 6:**
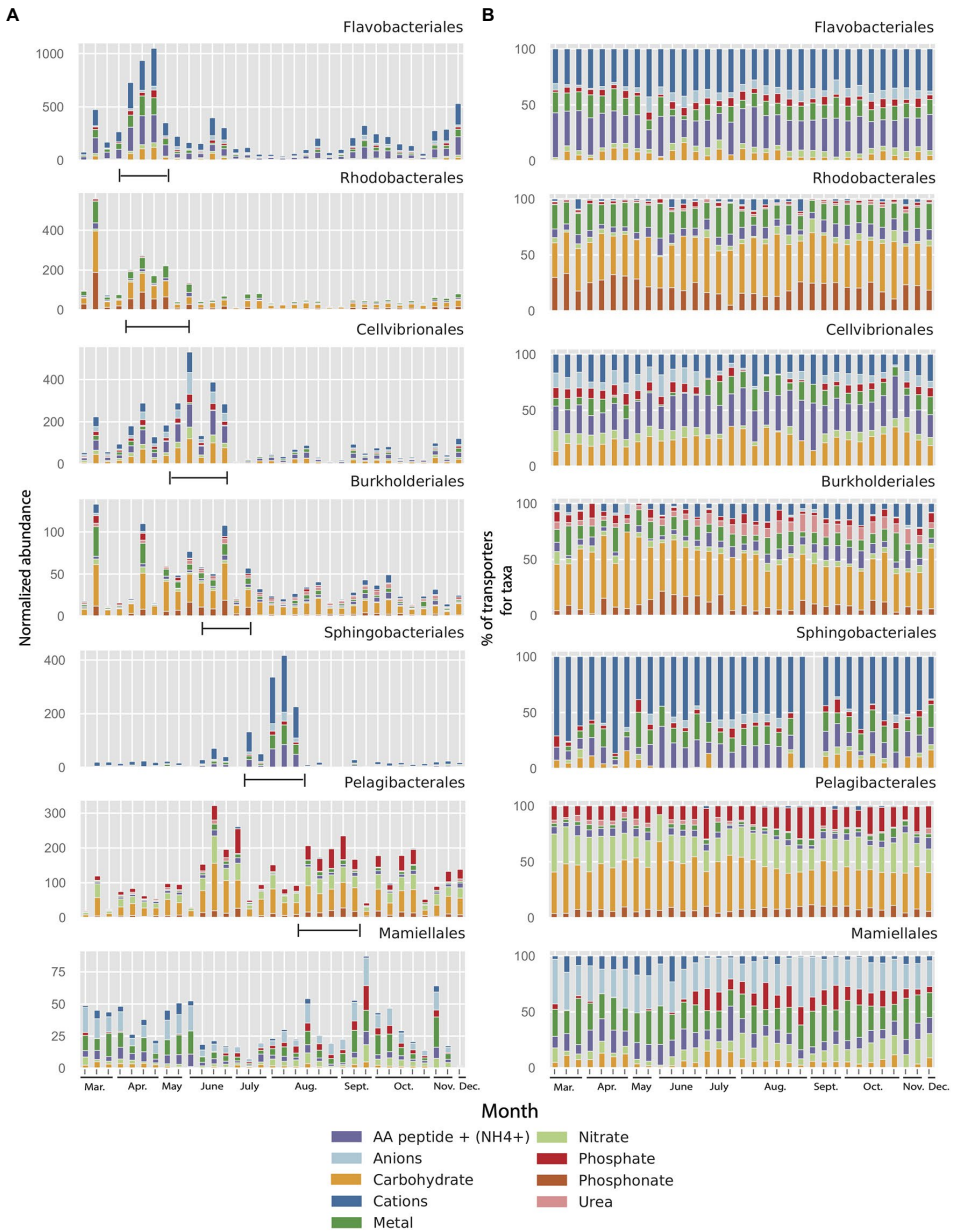
**(A)** Normalized abundance of transporter clusters related to different substrate categories and their affiliation to seven microbial taxon (order) in the LMO 2012 metagenome dataset (10months). Range mark in figure added manually to indicate the succession of transporter peaks. **(B)** Same data as in panel A but as percentages for each of the seven microbial taxa (order) in the LMO metagenome.

To analyze how differences in importers between taxa potentially are linked to changes in environmental conditions, we investigated the changes in relative abundance of importers in the seven most abundant orders during the 2012 growth period (10months, see Section “Materials and Methods” for details). This demonstrated that, most notably, the *Flavobacteriales* and *Sphingobacteriales* showed dramatic peaks in abundance of importers in the metagenome during the year (Figure 6A). In contrast, the *Pelagibacterales* showed much less variability in importer abundance during the 2012 growth period (10months). Interestingly, the peaks in importer abundance for the different taxa varied in a successional manner, starting at the time of the spring bloom with the orders *Flavobacteriales* and *Rhodobacterales* which peaked in mid-April and that had declined by mid-May. *Cellvibrionales* and *Burkholderiales* peaked later in June after which a pronounced peak in *Sphingobacteriales* could be seen ending the summer succession in early August (Figure 5A). From relatively lower abundances in spring, *Pelagibacterales* importers increased in early June and subsisted until the end of the year. The green algal order *Mamiellales* showed elevated levels of transporter proteins both during spring and autumn.

In spite of the large variation in the abundance of transporters between orders in the MG dataset (Figure 6A), the substrate specificity of the importers within each order remained remarkably stable over the year (Figure 6B). Equally striking were the order-specific differences in substrate affinity of the importers throughout the year. Accordingly, the dominant substrates affiliated with the *Flavobacteriales* were cations and amino acids/peptides, showing mean values over the entire sampling period of 50 and 27%, respectively (Figure 6B). Even though both *Flavobacteriales* and *Rhodobacterales* importers peaked during spring, the transporter composition clearly differed between the two orders. Whereas *Flavobacteriales* demonstrated more transporters for cations and amino acids/peptides, *Rhodobacterales* showed a more diverse distribution of importer proteins with less transporters for cations (18%) but rich in transporters for carbohydrates (36%), amino acids/peptides (19%), and phosphonate (19%). Also, the next two taxa in the succession of transporters, *Cellvibrionales* and *Burkholderiales* that followed in May and June, exhibited notable differences in transporter composition; primarily due to sizable portions of anion importers (26%) and amino acids/peptides importers (27%) in *Cellvibrionales* compared to high proportion of carbohydrate transporters (39%), but transporters for few amino acids/peptides (8%), cations (18%), and phosphonate (9%) in the *Burkholderiales*. Curiously, this distribution of transporters in *Burkholderiales* was rather similar to that of the *Rhodobacterales* that were dominant in early spring. Compared to the importer composition of the *Flavobacteriales*, the late summer-peaking Bacteroidetes order *Sphingobacteriales* demonstrated an even more pronounced dominance of cation transporters (70%) and a lower proportion of amino acids/peptides transporters. Compared to the other taxa, the *Pelagibacterales* were primarily characterized by a high proportion of transporters for phosphate (on average 15%) and nitrate (23%; Figure 6B). Interestingly, the proteobacterial *Rhodobacterales*, *Burkholderiales*, and *Pelagibacterales* had larger proportions of phosphonate transporters than the other taxa (mean 19, 9, and 7%, respectively). The *Mamiellales* stood out as having a particularly high proportion of transporters for anions (≥25%).

### Nitrogen and Phosphorus Importers

**Figure 7:**
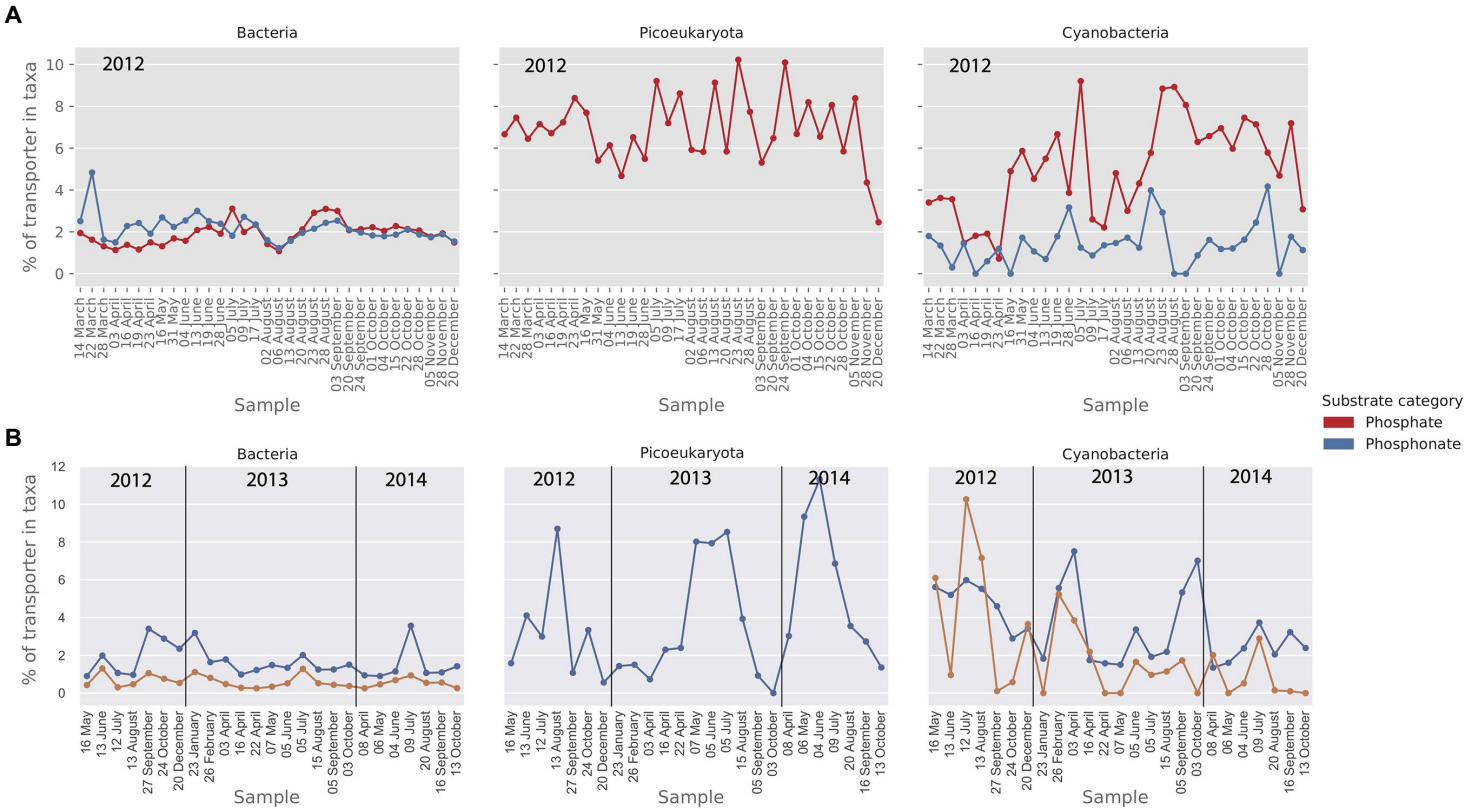
Relative abundance (MG) and activity profiles (MT) of transporters for phosphate and phosphonate in **(A)** the metagenomic dataset and **(B)** the metatranscriptomic dataset. Gene abundances and transcripts are shown relative to the total of the transporters within each taxonomic group. Only transporters that make up at least 1% of the relative transporter abundance/activity in one or more samples are shown here.

Uptake mechanisms for nitrogen and phosphorus are key traits for both heterotrophic bacteria and primary producers (Zweifel et al., 1993). To compare the prevalence and expression of those genes between bacteria, picocyanobacteria and picoeukaryotes, we grouped phosphorus and nitrogen importers together. The results are presented as the percentage of phosphorus and nitrogen importers relative to the 59 abundant importers identified in the metagenome and transcriptome datasets. Phosphate importers were found in all three plankton groups whereas phosphonate importers were only found in prokaryotes (Figure 7A). From the comparison in Figure 7A, it appears that phosphate transporters comprise a higher proportion (6%) of the importers in cyanobacteria compared to heterotrophic bacteria (2%). Phosphonate transporters, on the other hand, demonstrated similar levels in both cyanobacteria and heterotrophic bacteria (ca 2%). In the metatranscriptomic data, phosphonate also showed a similar relative importance in the cyanobacteria and bacteria at around 2%, whereas the higher gene abundance in cyanobacteria for phosphate importers seen in the MG data, was not seen in the MT (Figure 7B). The phosphonate importers were dominated by the genes *phnC*, *phnD*, and *phnE* found in the *phn* operon (Stosiek et al., 2019). The most abundant phosphate importer in bacteria, picocyanobacteria and picoeukaryotes belonged to the Pst-system, and the genes *pstC*, *pstA*, and *pstB* found in the *pstSCAB*-*phoU* operon were included in the analysis (Stosiek et al., 2019).

**Figure 8:**
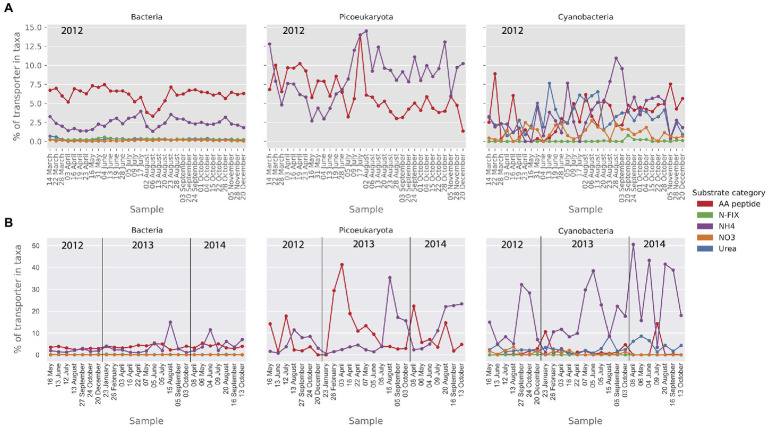
Relative abundance (MG) and activity profiles (MT) of selected mineral nutrient transporters within category nitrogen substrates in **(A)** the metagenomic dataset and **(B)** the metatranscriptomic dataset. Gene abundances and transcripts are shown relative to the total of the transporters within each taxonomic group. Only transporters that make up at least 1% of the relative transporter abundance/activity in one or more samples are shown here.

As for nitrogen importers, amino acid and ammonium transporters were abundant in the MG in all taxa (Figure 8A). The relative abundance of ammonium transporters was particularly high in the picoeukaryote MG data (up to 14% of transporters). The cyanobacterial community showed a wider nitrogen preference with a substantial presence of nitrate and urea transporters in the MG (Figure 8A). In the MT dataset (Figure 8B), the most notable observation was the high level of ammonium importer transcription in the cyanobacteria.

### Environmental and Metagenome Data

**Figure 9:**
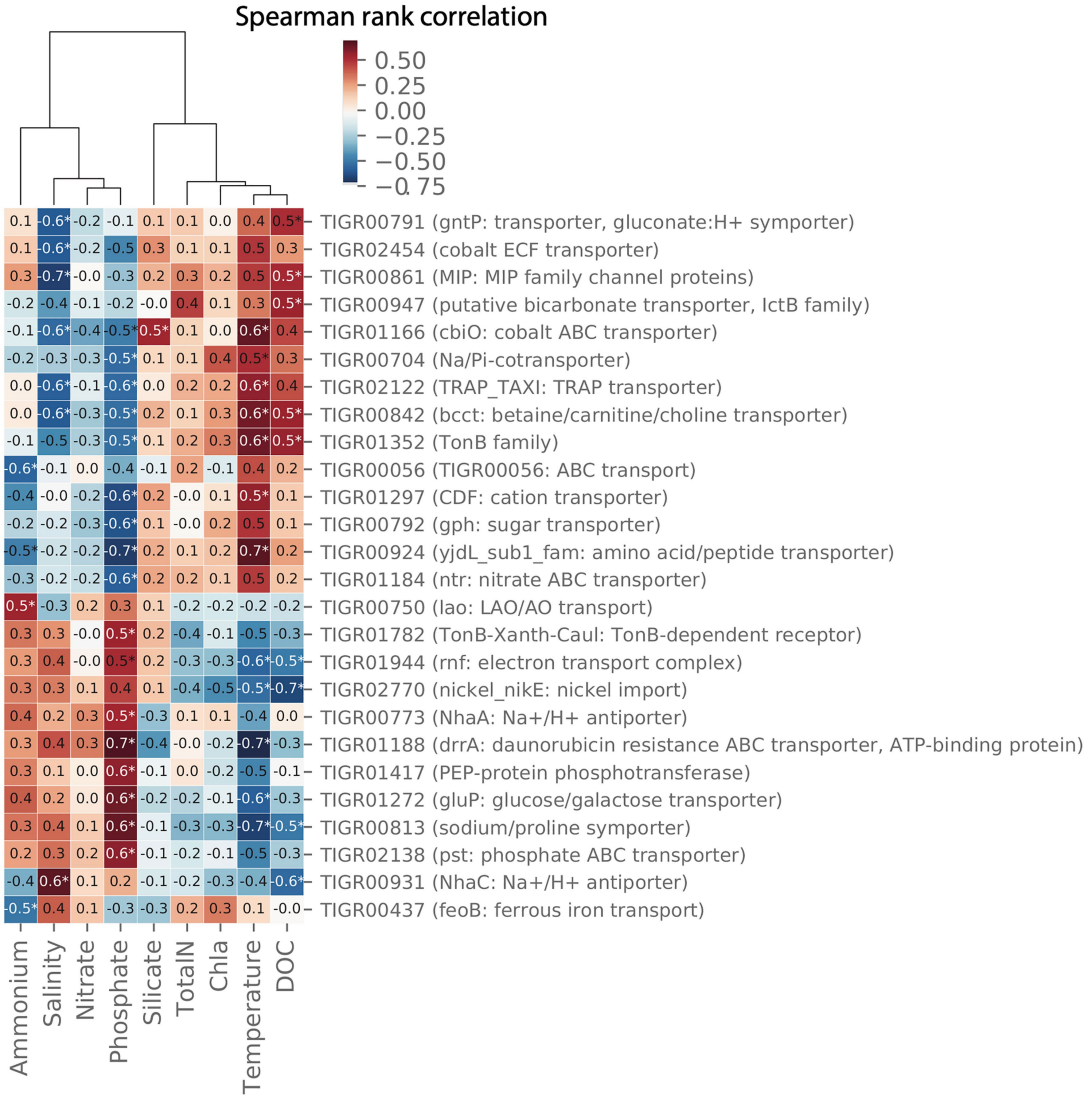
Clustermap showing pair-wise spearman correlation metrics for environmental data and transporters in the 2012 LMO metagenome (MG) dataset. Only transporters with at least pair-wise correlation with Spearman rank correlation coefficient (*ρ*)>0.5 and value of *p*<0.05 are shown.

Pair-wise spearman rank correlations between the abundance of importer proteins in the MG data set and environmental parameters measured at LMO are shown in the heat map in Figure 9. Two major groups of environmental variables were apparent with strong alternating negative or positive correlations to specific transporters: the first consisted of silicate, chlorophyll *a*, total nitrogen, temperature, and dissolved organic carbon (DOC); and the second of ammonium, salinity, phosphate, and nitrate.

For several transporters, the relative gene abundance was positively correlated to temperature and at the same time negatively correlated to phosphate. Three examples of proteins showing this pattern were (a) members of the solute:Na+ in symport (SSS) family of importers that have been suggested to transport sugars, amino acids, nucleosides, inositols, vitamins, and urea or anions depending on the system (Jung, 2002), (b), the cation diffusion facilitator (CDF) that is a broadly distributed family of transporters, and a number of which have been shown to transport divalent cations of cobalt, cadmium, and/or zinc (Paulsen and Saier, 1997), and (c) the Na+/H+ antiporter NhaC, involved in pH homeostasis and sodium extrusion (Ito et al., 1997).

### Exporters

The abundant exporter proteins found in the MG dataset (>0.5%) could be placed into two main groups (Table 2). The first group included 25 functionally quite diverse proteins. These include membrane translocation functions associated with structural elements, such as lipoproteins and lipopolysaccharides, in addition to proteins linked to efflux of mineral nutrients, such as nitrate. Also, a significant amount of transporter proteins related to protein trafficking were found and these included transporters of exoenzymes as well as pili components for adherence in biofilms and cell–cell contact. Compared to the number of specific importers in the MG data set, the group of export proteins were half as many, but clearly demonstrated a widespread potential for outward transport of material to the cell wall and exoenzymatic activities.

The second group of transporter proteins were comprised of secretion systems for toxins typically characterized as pathogenicity traits (Persson et al., 2009; Table 4). These proteins equaled one-third of the number of importers in the MG. In the LMO metagenome, transporters characterized as responsible for protein secretion was abundant throughout the 2012 growth season, with a tendency for elevated levels during algal bloom periods (corresponding to high Chla concentrations, see Figure 1). As an example, the abundance of the transporter family HlyIII peaked 1week after the Chl*a* spring peak and again during the autumn bloom (data not shown). This protein family shows high similarity to the pathogenicity trait expressed by *Bacillus cereus*, where the responsible gene has been shown to function as a channel-forming cytolysin (Baida and Kuzmin, 1996). Monomeric autotransporters were found in high abundance in the LMO MG. This kind of transporter molecule was the first type V secretion system studied in detail and importantly many virulence factors belong to this family (Meuskens et al., 2019).

**Table 4:** Exporter proteins related to common secretion types seen in pathogenic bacteria. Numbers refer to the total number of protein clusters in each secretion type.

Secretion type	Exporter proteins; toxin secretion						
	Type 1	Type II	Type III	Type V	Type VI	Other	Total
Protein clusters total numbers	13	20	2	8	27	4	74

## Discussion

Transporter proteins are involved in most aspects of microbial ecophysiology. Our study was therefore focused toward three ecological questions: bacterial succession, virulence, and biogeochemical indicators. As such, the results from frequent sampling of the LMO metagenome reported here demonstrated the fundamental relevance of transporter proteins in microbial ecology.

The most important result was a striking pattern that emerged in the distribution of transporters among different major taxa (Figure 6B). Despite dramatic environmental variation during the growth periods, the distribution of transporter proteins was similar in 33 metagenome samples. This was observed although the distribution was related to nine different substrates in seven microorganism orders, with changing cell abundance. It seems highly unlikely that this could be the result of random circumstances. The remarkable consistency of the substrate affinity distribution at a high phylogenetic level (order) suggests that transporter proteins may be viewed as central elements in a core genome characterizing each bacterial order. In addition, we came across several interesting transporter proteins that are exciting candidates for future ecological studies and these are briefly discussed below.

### Species Succession and Temporal Substrate Make-up

The spatiotemporal variation in bacterial community composition in the Baltic Sea has been studied extensively (Hagström et al., 2000; Riemann et al., 2008; Andersson et al., 2010; Dupont et al., 2014; Lindh and Pinhassi, 2018). In these and other studies, seasonal succession of bacterioplankton is strongly associated with temperature, followed by nutrient composition and accompanying microbes. Together, these factors explain most of the proliferation of abundant OTUs (Fuhrman et al., 2015; Sala et al., 2020). Notably, it has been stated that temperature changes can result in dramatic responses in the marine microbiome that in turn might influence the biogeochemistry of the habitat (Sjöstedt et al., 2012; Bergauer et al., 2018). This brings us to the question of how seasonal variation in temperature, environmental substrate composition, and species succession may be coupled. We can envision two different but simultaneously acting processes that influence the distribution of bacteria in temperate ocean surface waters (Hagström et al., 2017). In the first, as temperature changes with increasing insolation, the overall food web composition is modified and thus the substrate composition. This gives opportunities for additional taxa, carrying supplemental traits suited to exploit the new aspects of the environment, to emerge. In the second simultaneous process, following temperature as the driving force, bacterial strains can be expected to portray different temperature optima that will allow competition with varying success (Hagström et al., 2017). Each small temperature range would thus be occupied by strains carrying a core genome where many genes have been optimized through adaptive selection to operate efficiently at the optimum temperature, yet not being an excessive burden during temperatures outside the optimum. The resulting species composition and chains of succession would hereby be the result of a complex interplay between physicochemical conditions. In this scenario, although physically not separated in surface water, factors, such as insolation and temperature, would create ecological niches separated in time with large enough differentiation that each time period can be identified through its selective forces and thus generate a successional event. In this study, we determined how the diversity and relative abundance of transporter proteins in distinct bacterial taxa changed across the 2012 growth periods spring to autumn. The LMO dataset demonstrated transporter proteins linked to nine substrate categories that were limited to around 10 taxa (orders) for the bacteria domain (Figure 4). This finding is consistent with the results showing limited niche richness using a global co-occurrence network and calculating correlation scores between the abundances of marine microorganisms in 180 metagenomes (Coutinho et al., 2015). In that study, 297 organisms were found, and these were segregated into 11 major groups that occupy distinct ecological niches. Given that each major taxon in the LMO data could be linked to transporters related to each of nine different substrate categories, this would imply a maximum of about 100 taxa to be responsible for the transporter functionality.

We thus conclude that a limited number of bacterial orders seems to be able to provide a substantial diversity of transporter functionality that make them particularly successful during different seasons of the year. Having stated this, we do realize that taxonomy is not fully resolved at the genome scale, so inferring the number of taxa responsible for transporter functionality could be linked with uncertainties.

The spring bloom period is an obvious starting point for interpreting patterns in the annual succession in the Baltic Sea as in most temperate waters. The no-growth winter period is followed by conditions with increasing light and temperatures, resulting in a diatom and dinoflagellate spring bloom (Tamminen, 1995). These spring algae consume the mineral nutrients accumulated during winter, and a large phytoplankton biomass is amassed. The resulting large buildup of particulate and DOC represents energy that ultimately will be dissipated through heterotrophic (primarily bacterial) growth during the coming summer period (Zweifel et al., 1993; Thingstad et al., 2003). At the LMO station, the first bacterial successional event coinciding with the phytoplankton spring bloom is a rapid increase in the abundance of bacteria from the *Bacteroidetes* phylum with a peak from early-April to mid-May (Figure 2; Hugerth et al., 2015; Lindh et al., 2015). Typically, *Bacteroidetes* are early responders during phytoplankton blooms (Buchan et al., 2014), and the first peak in transporter proteins coincided with the spring bloom with importers related to the substrate categories cations and amino acids/peptides, phylogenetically linked to the order *Flavobacteriales* (*Bacteriodetes*). The substrate group “cations” notably includes several metal ions, but in relation to the spring bloom, most importantly, transport a broad variety of charged organic carbon compounds *via* the TonB dependent transporters (TBDTs).

To underscore this notion, TonB-linked outer membrane proteins, SusC/RagA/OmpW (Foley et al., 2016), showed high abundance for the sampling dates March 19 and 23 (data not shown). This class of importers likely facilitates the trans-localization of complex and high molecular weight substrates (Schauer et al., 2008). Transporter complexes, including outer membrane proteins, such as RagA and SusC, are likely to facilitate import of large degradation products of proteins or carbohydrates, respectively (Shi et al., 2007). Interestingly, previous studies have reported peaks of bacteria during phytoplankton blooms that possibly utilize TBDTs to take up carbon released from lysed algae (Teeling et al., 2012; Williams et al., 2013). Adding to the description of the spring bloom dynamics at LMO, uptake of smaller molecular weight substrates, such as glucose, acetate, pyruvate, leucine, and amino acids has been demonstrated to be low during spring as compared to the summer months (Bunse et al., 2019). This may be seen as an indication that enough rich complex organic substrates are available for the bacteria to ignore monomers at low (nanomolar) concentration. This is a situation that resembles catabolite repression (Stulke and Hillen, 1999).

Taken together, our results show differences in transporter cluster abundances related to phylogenetic affiliation across growth seasons. This would suggest that most major types of transporters are present at any given time, but that the distribution pattern stems from different taxa, mostly bacterial groups being dominant at different times of the year. The succession of transporter proteins with clear taxonomic links discussed above is difficult to explain by any other mechanism than a bacterial species succession during the growth period. We therefore find it reasonable to conclude that the substrate affinity traits that are revealed in this study represent a major mechanism explaining bacterial succession in nature.

### Bacteria Carry Transporters Involved in Virulence to Attack Neighboring Cells

Mechanisms for secretion of toxic substances have been studied extensively in relation to pathogenicity and human health, and it is suggested that aquatic bacteria are actively using such mechanisms for killing neighboring cells in the marine environment (Mayali and Azam, 2004; Persson et al., 2009; Rinta-Kanto et al., 2012; Teeling et al., 2012). Yet, it remains largely unexplored to what degree bacteria in nature carry and employ transporters that furnish virulence traits. When in the 2012 LMO samples, the average number of transporters responsible for toxin secretion was compared to the average number of substrate importer clusters, the ratio was 1:3. Also, the average abundance of each of the toxin secretion proteins amounted to about half of that found among the substrate importers. Thus, since the composition of the different secretion systems (Table 4) and their toxins were those typically described in medical textbooks, we conclude that the cell killing capacity has the potential to be ecologically significant. The secretion proteins were abundant throughout the growth season with a tendency to elevated levels during algal bloom periods, as was the case for the transporter protein family hlyIII that peaked a week after the chlorophyll spring peak. This protein family shows high similarity to the pathogenicity trait expressed by *Bacillus cereus*. In *Bacillus cereus*, the responsible gene has been shown to function as a channel-forming cytolysin (Baida and Kuzmin, 1996).

The type V secretion system, often called monomeric autotransporters (ATs), showed the highest abundance in the LMO MG data set as well as the highest activity (transcription) in the metatranscriptome (see Supplementary Table S1 for info on abundance and activity). In the simplest case, the type V system consists of only one polypeptide chain with a barrel translocator domain in the membrane and an extracellular passenger or effector region (Meuskens et al., 2019). The “passenger” can have many shapes and functions, such as the IgA protease from *Neisseria meningitidis* (Baida and Kuzmin, 1996), the adhesin involved in diffuse adherence-I from *Escherichia coli* (Meuskens et al., 2019) and pertactin from *Bordetella pertussis* (Mougous et al., 2006) among them. This transporter protein, with diverse functions frequently related to pathogenesis, deserves attention in future studies.

Another interesting secretion protein was EvpB/VC, a representative of the type VI secretion system that is part of the virulence arsenal of common pathogenic bacteria (Mougous et al., 2006). An example is the type VI secretion system related to the virulence locus (HSI-I) of *Pseudomonas aeruginosa* that encodes a protein secretion apparatus. Upon infection in humans, the substances secreted through this mechanism play a role in the pulmonary disease cystic fibrosis. Interestingly, HSI-I–related loci are widely distributed among bacterial pathogens and may play a general role in mediating host interactions (Mougous et al., 2006). In addition to being pathogens toward fish and zooplankton that do get infected (Guerrero et al., 1986; Rehnstam et al., 1989), the predatory role of bacteria with virulence traits against single cell eucaryotes constitutes an alternative life strategy for bacteria in the aquatic food web that can provide an enhanced flexibility in the uptake of organic substrates (Hagström et al., 2017).

### Transporter Proteins Give Clues to Prevalent Baltic Biogeochemistry

The Baltic Sea suffers from widespread eutrophication and high riverine input of allochthonous organic carbon (Elmgren et al., 2015). A significant part of its nitrogen and phosphorus pool is in organic form. Treatment of the organic nutrient pools is one of the structural uncertainties in current Baltic Sea biogeochemical models (Meier et al., 2019). Thus, understanding the biogeochemical processing of carbon, nitrogen, and phosphorus is important for guiding marine management on necessary mitigation measures. Since the rates of mineral nutrients that flow through the marine system is difficult to determine from nutrient concentrations, we looked closer at the abundance of importer proteins related to phosphorus, nitrogen, and carbon to see if they can give clues to the role of bacteria, cyanobacteria, and picoeucaryotes in the transformation on nutrients.

For phosphorus, these analyses were uncovered a surprising abundance of phosphonate transporters, in addition to the traditionally recognized phosphate transporters. Phosphonate has not received much attention as a potential P-source in the Baltic Sea but see Teikari et al. (2018). Phosphonates are found in various forms, such as phosphonolipids and as side groups on exopolysaccharides or glycoproteins, and can make up 25% of the 1–100nm size fraction of dissolved organic phosphorus (Sannigrahi et al., 2006). Phosphonate degradation is only known to be carried out by proteobacteria and cyanobacteria (Ilikchyan et al., 2009; Teikari et al., 2018). *Nodularia spumigena* (order *Nostocales*) is one of the dominant bloom-forming cyanobacteria in the Baltic Sea and has recently been found to carry phosphonate degrading genes (*phn*; Teikari et al., 2018). Still, the majority of filamentous cyanobacteria, including *N. spumigena*, would have been excluded in our MG and MT datasets due to filter size selection (3.0–0.2μm). This suggests that also at least some picocyanobacteria carry the genes for phosphonate uptake. The abundance and transcription of transporters linked to phosphonate uptake in both bacteria and cyanobacteria in the MG and MT data thus indicates that phosphonate uptake and transformations could be an important but largely overlooked component of P cycling in the Baltic Sea.

With regard to nitrogen, the abundance and expression of transporters at the LMO study site indicated that bacteria, picoeukaryotes, and picocyanobacteria all relied on uptake of both amino acids and ammonium and for cyanobacteria also urea. Abundance and expression of NO_3_ importers was consistently low, in accordance with more energy demanding uptake of NO_3_ compared to ${\mathrm{NH}}_{4}^{+}$. Ammonia and urea can cross cell membranes through passive diffusion but only when concentrations are high, and actually the expression of transporters involved in active uptake of these substrates is repressed when the concentrations of these compounds are elevated (Beckers et al., 2004; Kim et al., 2012). During summer, the predominant form of nitrogen uptake in the picoplankton size range at the LMO station was the ammonium ion (${\mathrm{NH}}_{4}^{+}$), which requires active transport across membranes. Since urea concentrations in the Baltic Sea are typically <0.5μmN (Sahlsten and Sörensson, 1989), the uptake of both urea and ammonium can thus be anticipated to be active (Solomon et al., 2010). Active ammonium transport is mediated by the Amt protein family which is ubiquitous and found in all domains of life (Wirén and Merrick, 2004) and high abundance and activity was detected in both the MG and MT data sets. Surprisingly, the cyanobacteria showed a very high, at times 50%, activity of ammonium transporters in the MT data set. In the prevailing model used to support management of nutrient input to the Baltic Sea, nitrogen fixation is activated when inorganic N:P ratios are below 16 and temperatures are above 14°C (Gustafsson et al., 2017). This can be compared to a N:P ratio of ≈7 at LMO during the summer months.

For carbon compounds, an important group of importers observed in the LMO data set was the TRAP-type transporter family. The TRAP substrates are diverse but share the feature that they are all organic acids (Mulligan et al., 2011). These importers made up 72% of the importer proteins identified in the carbohydrate group. As a comparison, transcripts of TRAP importers were found to be elevated during a phytoplankton bloom in the Gulf of Mexico (Rinta-Kanto et al., 2012). Another abundant transport family potentially involved in carbon compound uptake was the divalent Anion:Na+ Symporter (DASS) Family representing a large fraction of the anion transporters over the year. Functionally characterized proteins in this family transport inorganic sulfate and phosphate, but others transport organic di- and tricarboxylates as well as dicarboxylate amino acids.

TonB-dependent transporters are important for active uptake across the outer membrane in gram-negative bacteria (Schauer et al., 2008) and the LMO metagenome demonstrated that almost half (41%) of the relative abundance of the cation substrate group came from TBDTs (Supplementary Table S1). These proteins bind and transport ferric chelates (siderophores), as well as vitamin B12, nickel complexes and carbohydrates. These TonB-mediated transport processes require energy and a complex of inner membrane proteins, among them TonB, transduces energy to the outer membrane (Noinaj et al., 2010). The precise mechanism for this energy transfer is still unclear but it is derived from the proton motive force (Gresock and Postle, 2017).

For future management of the Baltic Sea, the specific transporter proteins discussed in this section represent possible indicators for the assessment of good environmental status according to the EU Marine Strategy Framework Directive (European Parliament, 2008). Measurements of the abundance of these proteins through a monitoring program may prove valuable data for the validation of biogeochemical models. Since the turnover of bacteria is rapid in the sea surface, as soon as growth of the population slows down, bacteria will effectively be removed, i.e., reduced to low numbers through grazing. Thus, we can expect the transcription of transporter proteins to be tightly coupled to the abundance of the genes.

### On the Ecology of “Metal” and “Other” Transporters

The metal ion transporter group of nine proteins (Table 2) represents a vital function in relation to essential metal cations, such as copper, iron, zinc, cobalt, nickel, and manganese. Since these transporters often are bidirectional, it is difficult to sort metal ion transporters into importer or exporter groups and we therefore kept them separated. Many metal functions are as important cofactors for enzymes. However, when present at high concentration, along with non-essential metals, such as cadmium, mercury, silver, and lead, essential metals can become toxic, since they can cause oxidative damages or compete with other essential ions (Nies, 2003). To counteract accumulation, metal ions are constantly transported in and out of the cell, for example by the CDF family of transporters (Montanini et al., 2007; Kolaj-Robin et al., 2015). CDF proteins share a two-modular architecture consisting of a transmembrane domain and a C-terminal domain and these were found in high abundance in our datasets. Bacterial CDF’s are primarily involved in metal tolerance/resistance and homeostasis by efflux of divalent metal cations from the cell (Kolaj-Robin et al., 2015; Chandrangsu et al., 2017). Among transporters classified as “others,” the six sub-unit gene complex *rnfABCDGE* that encodes an NADH oxidoreductase responsible for electron transport to the nitrogenase was particularly interesting since it is necessary for nitrogen fixation (Koo et al., 2003). At the LMO sampling site, the *rnfABCDGE* genes are found in relatively low abundance, but N_2_ fixation is important in this brackish sea area – thus, these genes are ecologically highly relevant. From these examples, it appears that detailed future studies of eco-physiological adaptations to metals and particular transport systems will greatly illuminate the narrative of the microbial world.

### Take Home Message for Modelling

In this study, we have provided information linking microbial growth dynamics to the biogeochemistry of dissolved organic and mineral nutrients in marine surface waters. Based on the fundamental observation, that flow of matter between stocks in biogeochemical models is mediated by transporter proteins at the cell surface and not biochemistry within the cell, transporter proteins would be the obvious link between metagenome data and model output. Noteworthy, the prevalence of the transporter proteins recorded in the Baltic metagenome was seen to trace the seasonal bacterial succession. This connection may be important as *in situ* metagenome data could potentially serve to validate future biogeochemistry models, in addition to the present use of inorganic nutrient data. But in order to compute these dynamics in detail, a realistic simulator would be needed. This will require basic information, such as transporter protein diversity and functionality, along with rules for organism encounters, to set limits for the system. We suggest future work may be directed toward establishing a “molecular simulator” that would serve to inform on substrate affinities of transporters in relation to microbial community dynamics. We think that the limited diversity and the consistency of the molecular traits involved in substrate transport, as demonstrated in this paper, may be findings that could be used to estimate costs and benefits of different life strategies, as demonstrated in earlier modelling work (Blackburn et al., 1996, 1997, 1998).

## Data Availability Statement

Publicly available datasets were analyzed in this study. These data can be found here: Baltic Sea Reference Metagenome (BARM), Alneberg et al., 2018b, available at: https://github.com/ChristoferLNU/transporters/blob/master/results/transport-clusters.2017_12.tab.

## Author Contributions

ÅH, UZ, JSu, and BM contributed to conception and design of the study. JSu, UZ, ÅH, and CO performed the bioinformatic analysis and organized the database. CO, CB, and JSj performed field work and laboratory analysis at the LMO sampling site. ÅH, JSu, and UZ wrote the first draft of the manuscript. JP, ÅH, and CO did the final editing. ÅH and JP were responsible for funding acquisition. All authors contributed to manuscript revision, read, and approved the submitted version.

## Funding

*Via* the BONUS BLUEPRINT project, ÅH, JP, and BM received funding from BONUS, the joint Baltic Sea research and development program (Art 185), and the Swedish research council FORMAS. Funding for field sampling and sequencing was also provided through the Swedish governmental strong research programme EcoChange to JP. JSu received funding from the Knut and Alice Wallenberg Foundation. CB was financially supported by HIFMB, a collaboration between the Alfred-Wegener-Institute, Helmholtz-Center for Polar and Marine Research, and the Carl-von-Ossietzky University Oldenburg, initially funded by the Ministry for Science and Culture of Lower Saxony and the Volkswagen Foundation through the “Niedersächsisches Vorab” grant program (grant number ZN3285).

## Conflict of Interest

The authors declare that the research was conducted in the absence of any commercial or financial relationships that could be construed as a potential conflict of interest.

## Publisher’s Note

All claims expressed in this article are solely those of the authors and do not necessarily represent those of their affiliated organizations, or those of the publisher, the editors and the reviewers. Any product that may be evaluated in this article, or claim that may be made by its manufacturer, is not guaranteed or endorsed by the publisher.

## Footnotes

^1^ http://www.uniprot.org

^2^ http://operondb.cbcb.umd.edu
